# *In vitro* Evaluation of the Remineralizing Potential and Color Change of Modified Fluoride Varnish with Polyvinylpyrrolidone-Coated Silver Nanoparticles on Incipient Enamel Lesions in Primary Teeth 

**DOI:** 10.30476/dentjods.2025.107027.2711

**Published:** 2026-09-01

**Authors:** Faranak Farahmand, Azade Rafiee, Najmeh Mohammadi

**Affiliations:** 1 Student Research Committee, Dept. of Pediatric Dentistry, School of Dentistry, Shiraz University of Medical Sciences, Shiraz, Iran.; 2 Oral and Dental Disease Research Center, Dept. of Pediatric Dentistry, School of Dentistry, Shiraz University of Medical Sciences, Shiraz, Iran.; 3 Pediatric Dentistry, Private Practice, Shiraz, Iran.

**Keywords:** Silver Compounds, Fluorides, Nanoparticles, Tooth Remineralization, Microscopy

## Abstract

**Background:**

Dental caries is closely associated with biofilms and characterized by cycles of demineralization and remineralization that weaken tooth structure over time. Given the need for effective prevention and enhanced remineralization, developing a more esthetic and efficient approach is essential in improving oral health outcomes.

**Purpose:**

The present study aimed to assess the remineralizing potential and color change of modified fluoride varnish with polyvinylpyrrolidone-coated silver nanoparticles (PVP-coated AgNPs) on incipient enamel lesions in primary teeth.

**Materials and Method:**

In this *in vitro* study, 130 caries-free primary canine teeth underwent caries induction and were randomly assigned to one of three treatments: (1) fluoride varnish (FV), (2) silver diamine fluoride (SDF), and (3) FV with PVP-coated AgNPs. After thermocycling-aging and an eight-day pH cycling regimen, the remineralization effects and color change were evaluated by enamel microhardness (EMH) test and spectrophotometric color assessment. Ten samples were used for surface morphology assessment with field emission scanning electron microscopy and energy-dispersive X-ray spectrometry (FESEM-EDS). One-way ANOVA and Tukey's post-hoc tests were used for data analysis (*p* Value<0.05).

**Results:**

The highest average±SD recovery of EMH values was found in the SDF group (52.43±2.58), followed by FV with PVP-coated AgNPs group (43.21±1.73), and FV group (35.60±1.36), respectively (all, *p*< 0.001). The voids on the demineralized enamel surface were partially coated with newly deposited minerals in FESEM images of all groups. SDF Group had the best calcium/ phosphorus ratio among the intervention groups (2.58± 0.001); followed by FV with PVP-coated AgNPs group (1.94±0.001) and FV group (1.91± 0.001), respectively. The color change of enamel samples treated with FV and modified FV with PVP-coated AgNPs was clinically imperceptible (all, ∆E < 3.3), whereas SDF-treated samples caused distinct staining (*p*< 0.001).

**Conclusion:**

FV with PVP-coated AgNPs effectively remineralizes incipient enamel lesions in primary teeth with minimal color change, offering a promising alternative to SDF.

## Introduction

Dental caries, a prevalent biofilm-mediated disease, involves cyclic demineralization and remineralization that progressively degrades tooth structure [ 1 ]. Preventive strategies using fluoride-based and non-fluoride-based remineralizing agents are essential to inhibit caries progression and promote enamel repair [ 2 - 3 ]. Sodium fluoride varnish (FV) enhances remineralization by facilitating calcium fluoride deposition [ 4 ], while silver diamine fluoride (SDF) arrests caries through silver phosphate and calcium fluoride formation and antibacterial action [ 5 - 6 ]. However, SDF’s permanent black discoloration of enamel limits its esthetic acceptability [ 7 ]. Silver nanoparticles (AgNPs) are notable for their cost-effectiveness, low toxicity, high anticariogenic effects, and esthetic advantage of not causing discoloration in dental enamel compared to conventional silver products [ 8 ]. To address these drawbacks, AgNPs have been developed to enhance enamel remineralization and minimize staining; however, challenges such as AgNP long-term stability in oral environments and the need for clinical validation remain [ 9 - 10 ]. Surface capping enhances the biological properties of AgNPs by stabilizing them during synthesis [ 9 ]. Capping agents, such as polyvinylpyrrolidone (PVP), glycerol, polyvinyl alcohol, sodium oleate, and polyethylene glycol, regulate AgNPs' growth rate, prevent agglomeration, optimize their characteristics, and reduce their dimensions [ 11 ]. PVP, a natural polymer, is commonly used as both a reducing and capping agent, providing stability and enabling sustained drug release and wound healing without adverse effects [ 11 - 12 ].

Numerous studies have investigated the incorporation of AgNPs into dental materials, revealing their promising ability to inhibit caries progression and remineralize early enamel lesions [ 12 - 18 ]. An in vivo study has shown that AgNP-FV combinations enhance remineralization in primary teeth [ 12 ]. Nanosilver FV has been found to prevent white spot lesions during orthodontic treatment, indicating its potential in caries prevention [ 13 ]. A randomized controlled trial reported that nanosilver fluoride, combined with self-assembling peptides, promotes remineralization of early enamel caries lesions in primary teeth, outperforming FV in microhardness and lesion depth reduction [ 14 ]. *In vitro* studies have explored the remineralizing potential of nanosilver fluoride compared to SDF and FV in artificial caries models, yielding varied findings [ 15 - 16 , 18 ]. Nozari *et al* . [ 15 ] reported that nanosilver fluoride demonstrated the greatest remineralization efficacy compared to FV and nanohydroxyapatite serum in remineralizing initial caries in primary teeth. In contrast, Akyildiz and Sönmez [ 16 ] found that FV and SDF outperformed nanosilver fluoride in terms of Vickers microhardness values for post-demineralization enamel, with nanosilver fluoride showing lower effectiveness (FV: 229.96, SDF: 222.96, nanosilver fluoride: 191.36), suggesting it may not be a viable alternative to routine fluoride treatments without further investigation. Meanwhile, El-Desouky *et al* . [ 18 ] indicated that nanosilver fluoride exhibited a comparable effect to FV in limiting enamel demineralization under acidic challenge, positioning it as a potential alternative preventive agent in primary teeth. These conflicting results highlight the need for further research to clarify AgNPs' efficacy relative to SDF and FV. Additionally, higher concentrations of nanosilver fluoride have been effective in arresting caries in primary molars [ 17 ]. The use of PVP-coated AgNPs has been highlighted for enhancing stability and antibacterial activity, making them suitable for dental material integration [ 11 ].

This study addresses the gap in developing a remineralizing agent that combines the efficacy of FV with the esthetic benefits of PVP-coated AgNPs. The logic of this approach lies in leveraging fluoride’s remineralizing capacity, AgNPs’ properties, and PVP’s stabilizing role to enhance remineralization while minimizing discoloration, a key limitation of SDF. We hypothesize that the modified varnish will outperform FV in remineralization and SDF in esthetic outcomes for incipient enamel lesions in primary teeth. Using Vickers microhardness (a technique to measure enamel strength), field emission scanning electron microscopy with energy-dispersive X-ray spectrometry (FESEM-EDS, a method to visualize enamel surface and measure its mineral content), and spectrophotometry, this study was conducted to evaluate the remineralizing potential and color change of the modified varnish compared to FV and SDF. 

## Materials and Method

### Sample size calculation and sample selection

Based on the results of the study by Favaro *et al* . [ 19 ] and assuming an alpha of 0.05 and a power of 95%, the minimum sample size per group was calculated as 20 using the G*Power statistical software (version 3.1). After explaining the study purpose, privacy preservation, and data anonymity to the parents or guardians, written informed consent was obtained at the time of tooth extraction. In total, 142 intact primary canine teeth were obtained for indications related to orthodontic treatment by a pediatric dentistry resident other than the researcher under supervision at the pediatric dentistry department of Shiraz Dental School. The study protocol was reviewed and endorsed by the University's Ethics Review Committee (IR.SUMS.DENTAL.REC.1402. 090). Preparation of the teeth for the experiment involved a meticulous cleaning, debridement, and disinfection process using a 0.1% chloramine T solution for a duration of one month. Thereafter, the teeth were stored in deionized water that was refreshed on a weekly basis until utilization for the experiment. The exclusion criteria included the enamel surfaces with structural imperfections, fracture lines, and stains upon examination at 20× magnification of a stereomicroscope (Motic K, Wetzlar, Germany) and baseline surface hardness values measuring below 250 Vickers hardness number (VHN) [ 20 ]. The roots were removed to one mm below the cementoenamel junction. Twelve teeth were excluded, and 130 teeth met the inclusion criteria.

### Sample preparation

The enamel blocks were prepared, as explained before by Rafiee *et al* . [ 6 ]. Briefly, the labial surfaces of the crowns were embedded horizontally in acrylic resin. The enamel surfaces were sequentially polished with waterproof silicon carbide papers with grit sizes of 600, 800, 1200, 2400, and 4000, followed by 1-μm aluminum oxide for a smooth finish. Next, the samples were thoroughly cleaned with deionized water for a period of 20 seconds. Thereafter, the samples were dried and coated with two layers of nail polish, leaving a 2×4mm exposed window on the most horizontal part of the labial surface. 

### Modified FV with PVP-coated AgNPs preparation

The method detailed in a previous study conducted by Haghgoo *et al* . [ 21 ] was used to prepare the modified FV. Briefly, 0.5 g of PVP-coated AgNP powder (Ag, 99.99%, 50-80 nm, w/~0.2% PVP) (US Research Nanomaterials, Houston, USA) was added to 10 ml of a 22600-ppm FV (FluoroDose®, Centrix Inc., Shelton, USA) under stirring at room temperature until total dispersion in a light-proof brown container, resulting in 5% PVP-coated AgNP concentration. 

### Early caries lesion induction

The demineralization protocol to create the incipient lesion consisted of placing each sample in 15 mL of the demineralizing solution containing 0.1 mM lactic acid solution, 3 mM CaCl, 3 mM KH_2_PO_4_, and 0.2% guar gum at 37˚C and a final pH of 4.5. The demineralization process lasted for 96 h, and the solution was replaced after 48 h [ 20 ].

### Group allocation

Of 130 selected teeth, 10 samples were used for FESE-M-EDS evaluation at baseline level (n=2), after demineralization (n=2), and after remineralization pertaining to each group of the study (n=6). The rest of the 120 teeth were arbitrarily allocated to three groups (n=40). Each group was randomly split into two sub-groups for enamel microhardness (EMH) (n=20) and color change (n=20) evaluation, using a random sequence generator. The groups were defined based on the treatment received as Group 1: FV, Group 2: SDF (positive control) and Group 3: FV with PVP-coated AgNPs.

For groups 1 and 3, a thin layer of FV (FluoroDose ®, Centrix Inc., Shelton, USA) or modified varnish with PVP-coated AgNPs was placed on the dry, exposed enamel surface with a clean microbrush for four minutes [ 20 ]. Samples of group 2 received 38% SDF solution (Caries arrest®, Dengen dental, India) for two minutes, during which it was agitated with a microbrush for one minute. The remaining unreacted SDF was absorbed using a cotton swab [ 6 ].

Immediately after the intervention, each sample was transferred to 15 mL of artificial saliva at 37ºC to promote ionic exchange with the enamel. The artificial saliva contained 0.2 mM glucose, 0.1 mM C_8_H1_5_NaO, 9.9 mM NaCl, 1.5 mM CaCl_2_.H_2_O, 3 mM NH_4_Cl, 17 mM KCl, 2mM NaSCN, 2.4 mM K_2_HPO_4_, 3.3 mM urea, 2.4 mM NaH_2_PO_4_, and 11 µM ascorbic acid (pH 6.8) [ 20 ]. After 24 h, the varnish coatings were meticulously removed with gauze soaked in a 50% acetone solution [ 4 , 20 ]. Then, the blocks were rinsed for 30 seconds with deionized water. 

### Thermocycling-aging and pH cycling of the specimens

To more accurately imitate the oral cavity conditions, each sample was subjected to thermocycling-aging (1000 thermal cycles, ranging from 5 to 55°C, a 30-second dwell time, a 15-second transfer time, and 2-week storage in deionized water) [ 6 ] and an eight-day pH cycling procedure [ 4 ]. The pH cycling was performed by placing each block in the demineralization solution for two hours and in the remineralization solution (1.5 mM CaCl_2_, 0.9 mM KH_2_PO_4_, 150 mM KCl, 0.05µg F/mL in 0.1 M Tris buffer, pH 7.0) for 22 h at 37ºC [ 4 ]. The solutions were refreshed on day four to prevent solution supersaturation [ 4 , 20 ]. The microhardness, morphology, and color changes were assessed on the eighth day [ 4 ].

### EMH assessment

The Vickers microhardness test was done on enamel samples at three stages including baseline (intact enamel), demineralized enamel, and after intervention for the three study groups. The Vickers hardness number (VHN), a measure of enamel surface strength, was determined by applying a controlled force with a diamond indenter to create a square-shaped indentation, with the VHN calculated from the indentation size to reflect the enamel’s resistance to deformation [ 22 ]. A universal testing machine (MHV-1000Z, SCTMC, China) was used to apply a 200g force for 15 s at five points, each spaced 100µm apart, to assess EMH values [ 6 ]. The following formula was implemented to calculate the percentage recovery of enamel microhardness (%REM) for each group:

$\mathrm{\%REM}=\frac{\mathrm{VHN of remineralized enamel}-\mathrm{VHN of demineralized enamel}}{\mathrm{VHN of sound enamel}-\mathrm{VHN of demineralized enamel}}\times \mathrm{100}$

### Surface morphology assessment 

The selected 10 samples were dehydrated and gold-sputtered in a vacuum evaporator for 1 min at 20 mA [ 20 ]. The morphological microstructure of the enamel surfaces was then analyzed using FESEM-EDS (SIGMA 500, ZEISS, Oberkochen, Germany) at an accelerating voltage of 5 kV and 15000× magnification level. 

The elemental compositions, including calcium (Ca), phosphorus (P), silver (Ag), and fluorine (F), were determined in weight (wt.%) using EDS at a beam voltage of 5 kV. The spatial distribution of the examined elements was visualized by EDS mapping.

### Color assessment 

L*a*b* values (Commission Internationale de l’éclairage) of the samples were recorded at baseline (intact enamel) after demineralization and remineralization. L* value measures brightness, ranging from 0 (dark) to 100 (bright). The a* value indicates the spectrum from redness (+a*) to greenness (-a*), while the b* value expresses yellowness (+b*) to blueness (-b*) [ 6 ]. 

We employed a 2×4mm-window silicone putty jig, corresponding to the exposed enamel area, to ensure precise repeated measurements. All the measurements were performed by one examiner using a spectrophotometer (Minolta Chromameter CR-241, Minolta Camera Co., Osaka, Japan) over a gray background (L* =49.2, a* =-0.4, b*=0.0). The color change (∆E) between the two stages was quantified based on the following equation: ∆E = √ (∆L*) ^2^+ (∆a*) ^2^+ (∆b*) ^2^. ∆E and ∆L were calculated for demineralization vs. baseline (∆E and ∆L _Dem-Base_), intervention vs. demineralization (∆E and ∆L _Int-Dem_), and baseline vs. intervention (∆E and ∆L _Int-Base_) stages. 

### Statistical analysis

All measurements were expressed as mean value ± the standard deviation (±SD) of the mean and analyzed using SPSS version 22 software (IBM, NY, USA). The normality of the data distribution was assessed with the Shapiro-Wilk test. The EMH, %REM, ΔE, and ΔL values were analyzed with one-way ANOVA and Tukey’s post-hoc test. Besides, repeated measure ANOVA and Sidak post-hoc tests were used for intra-group comparisons at different time points (sound, demineralized, and remineralized EMH values). The level of significance was established at 0.05 for all statistical analyses. 

## Results

The average ±SD for EMH values at three stages, as well as the %REM for each group, are demonstrated in Table 1. The baseline EMH values ranged between 339.87 and 352.73 VHN, averaging at 347.82±3.54 VHN. The differences among the groups were not statistically significant (*p*= 0.074). After demineralization, the EMH values decreased dramatically in all groups, ranging between 244.46 and 262.38 VHN (all, *p*< 0.001, averaging at 251.82±3.76), with no significant differences (*p*= 0.089). The application of remineralizing materials followed by thermocycling-aging and pH cycling resulted in increased EMH values compared to the demineralized status (all, *p*< 0.001). To better compare the EMH values at three time points, the %REM was calculated using the equation described previously. Despite degrees of recovery in EMH values in all intervention groups (all, *p*< 0.001), the highest %REM was seen in the SDF group (52.43±2.58), followed by FV with PVP-coated AgNPs group (43.21±1.73), and FV group (35.60±1.36).

**Table 1:** The enamel surface microhardness values (mean±SD) at baseline, demineralization, and remineralization stages, as well as the percentage recovery of enamel microhardness (%REM) among the study groups (n= 20)

Group	Baseline	Demineralization	Remineralization	%REM
FV	349.60±3.91 ^A, a^	253.14±4.67 ^A, b^	287.50±3.50 ^A, c^	35.60±1.36 ^A^
SDF	347.05±2.59 ^A, a^	252.14±2.98 ^A, b^	301.92±2.40 ^B, c^	52.43±2.58 ^B^
FV+PVP-AgNPs	346.85±3.48 ^A, a^	250.25±3.009 ^A, b^	292.00±3.71 ^C, c^	43.21±1.73 ^C^
*p* Value	0.074	0.089	<0.001	<0.001

In each row, means with the same lowercase letter are not significantly different (within-group analysis).

In each column, means with the same capital letter are not significantly different (between-group analysis).

Fluoride varnish (FV), silver diamine fluoride (SDF), fluoride varnish with polyvinylpyrrolidone-coated silver nanoparticles (FV+PVP-AgNPs), percentage recovery of enamel microhardness (%REM)

**Figure 1:**
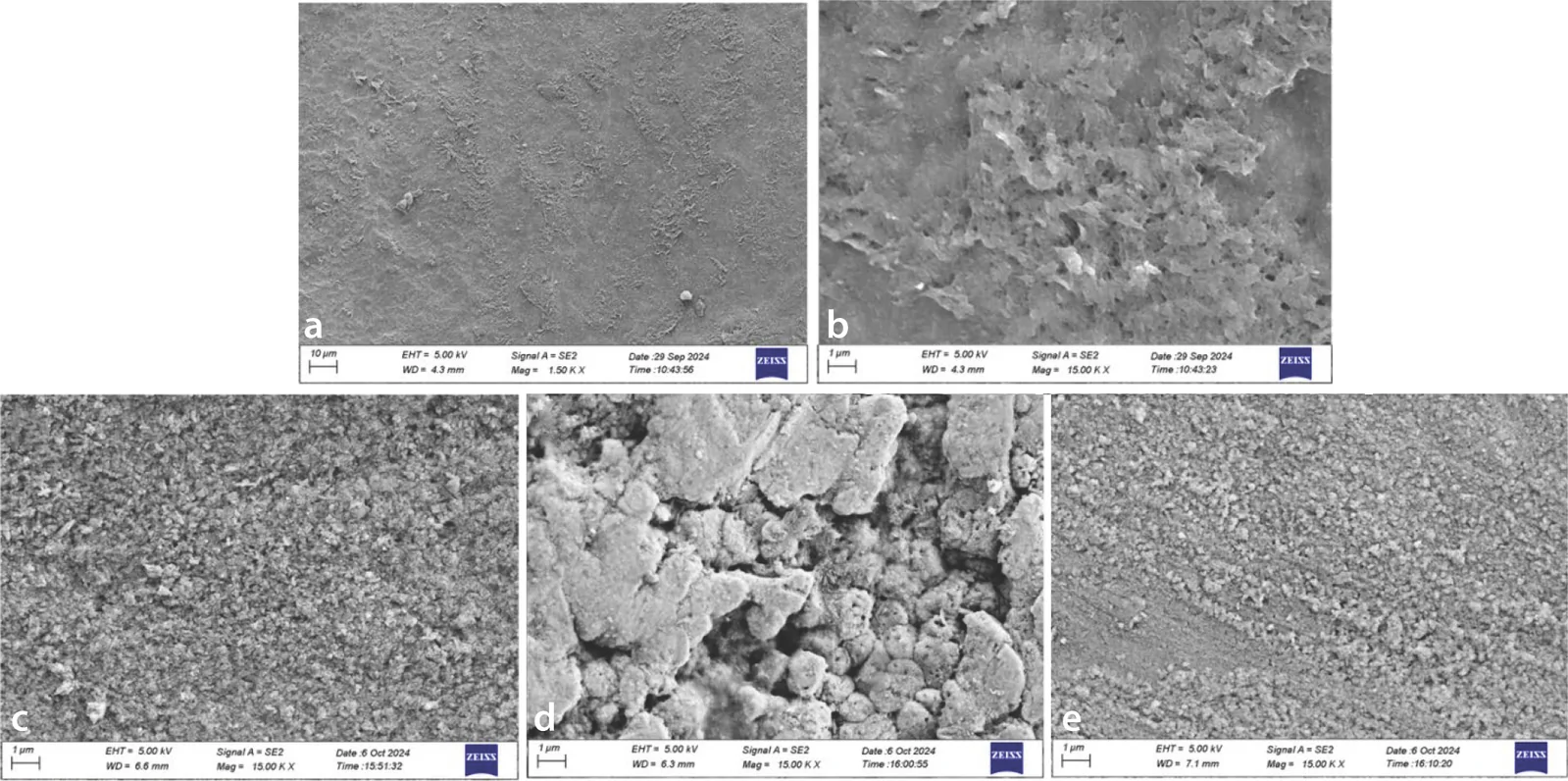
The FESEM images of the primary tooth enamel, **a:** At the baseline, **b:** After demineralization, **c:** Treated enamel surfaces with FV; **d:** Treated enamel surfaces with SDF; **e:** treated enamel surfaces with modified FV with PVP-coated AgNPs

The surface morphology at baseline, after demineralization, and after application of the remineralizing agents is represented in FESEM images (Figure 1a-e). The breakdown of normal enamel structure was evident in most areas subsequent to demineralization, a consequence of diminished organic and inorganic constituents (Figure 1b). The deposition of minerals in all groups following the intervention resulted in relative coverage of the voids caused by demineralization (Figure 1c-e). The findings validate that all intervention groups could assist in establishing mineral deposits on enamel surfaces. The elemental composition in wt.% for Ag, F, Ca, and P and the Ca/P ratio based on the EDS results are presented in Table 2. The Ca/P ratio of sound enamel was 2.08 ± 0.04, which decreased to 1.48±0.004 after demineralization. There was evidence of improvement in the Ca/P ratio following intervention and pH cycling. 

**Table 2:** The values of silver (Ag), fluoride (F), calcium (Ca), and phosphorus (P) elemental composition in weight (wt.%) and the Ca/P ratio of the enamel surface at baseline (sound enamel), after demineralization, and after intervention and pH cycling (n= 2)

Group	Ag	F	Ca	P	Ca/P ratio
Baseline	_	_	0.98 ± 0.003	0.47 ± 0.007	2.08 ± 0.004
Demineralization	_	_	20.55 ± 0.07	13.49 ± 0.014	1.48 ± 0.004
FV	_	0.04 ± 0.004	27.35 ± 0.003	14.25 ± 0.002	1.91 ± 0.001
SDF	70.27 ± 0.014	0.09 ± 0.003	0.8 ± 0.004	0.31 ± 0.007	2.58 ± 0.001
FV+PVP-AgNPs	14.99 ± 0.007	0.06 ± 0.004	28.2 ± 0.003	14.48 ± 0.007	1.94 ± 0.001

Silver (Ag), fluoride (F), calcium (Ca), phosphorus (P), Fluoride varnish (FV), silver diamine fluoride (SDF), fluoride varnish with polyvinylpyrrolidone-coated silver nanoparticles (FV+PVP-AgNPs)

**Figure 2:**
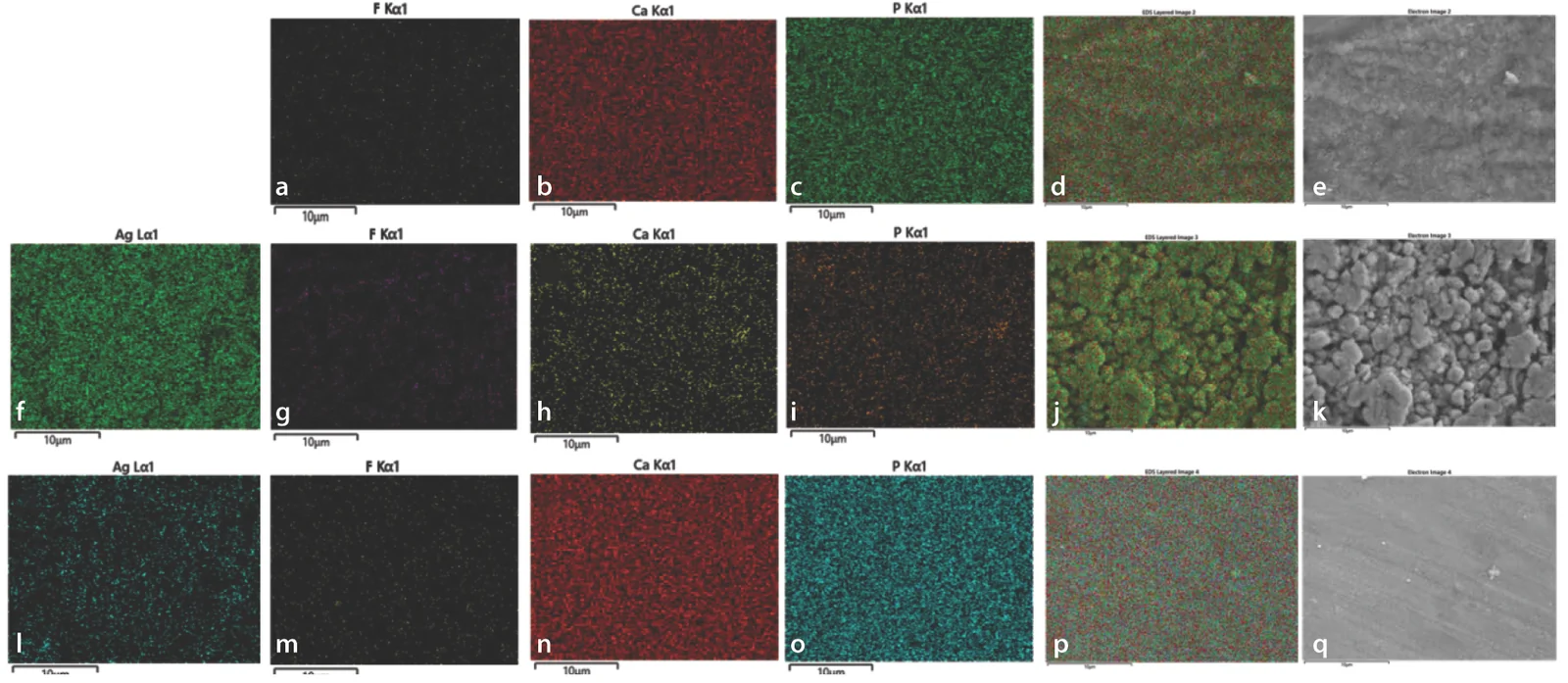
The FESEM-EDS elemental mappings of enamel surfaces treated with FV (a-e), SDF (f-k), modified FV with PVP-coated AgNPs (l-q). The distribution pattern for silver (f and l), fluoride (a, g, and m), calcium (b, h, and n), and phosphorus (c, i, and o) elements, the collective distribution (d, j, and p), as well as the corresponding FESEM images (e, k, and q), are demonstrated

The best value of the Ca/P ratio in the treated enamel surfaces was found in the SDF group (2.58±0.001), followed by FV with PVP-coated AgNPs group (1.94± 0.001), and FV group (1.91±0.001). The SDF-treated surfaces exhibited the greatest levels of F and Ag among all groups. Figure 2 illustrates the EDS-elemental mapping of Ag, F, Ca, and P elements in each intervention group. 

The average ± SD values of ∆L and ∆E were determined using the previously explained formula. A shift towards the lighter colors in demineralized samples was observed compared to their baseline attributes (Table 3). A significant discrepancy was observed in the values of ΔE and ΔL among all groups after the intervention compared to demineralization or baseline stages (∆E and ∆L _Dem-Base_ or ∆E and ∆L _Int-Dem_) (all, *p*< 0.001). The SDF-treated surfaces exhibited the highest degree of color change, with the darkest colors. 

**Table 3:** Color change (ΔE) and brightness change (ΔL) mean ± standard deviation (SD) at different time points among the intervention groups (n=20)

Group	Dem. vs. Base.		Int. vs. Dem		Int. vs. Base.	
	∆E	∆L	∆E	∆L	∆E	∆L
FV	4.32±0.460^A^	4.18±0.487^A^	3.85±0.533^A^	-3.77±0.579^A^	1.39±0.288^A^	0.41±0.29^A^
SDF	4.431±0.365^A^	4.36±0.433^A^	26.82±1.76^B^	-26.56±1.68^B^	22.52±1.82^B^	-22.20±1.71^B^
FV+PVP-AgNPs	4.38±0.275^A^	4.36±0.278^A^	6.67±0.49^C^	-6.73±0.62^C^	3±0.325^C^	-2.48±0.66^C^
*p* Value	0.723	0.388	<0.001	<0.001	<0.001	<0.001

In each column, means with the same capital letter are not significantly different (between-group analysis).

Demineralization (Dem.), baseline (Base.), intervention (Int.), color change (ΔE), brightness change (ΔL), Fluoride varnish (FV), silver diamine fluoride (SDF), fluoride varnish with polyvinylpyrrolidone-coated silver nanoparticles (FV+PVP-AgNPs)

## Discussion

Despite recent advances, dental caries continues to be a major health problem [ 23 ]. Therefore, evaluating potential remineralizing agents for carious lesions remains a key focus for researchers. The current study aimed to assess the remineralization potential of modified FV with PVP-coated AgNPs on demineralized primary tooth enamel.

Considering the reliability and non-destructiveness of the Vickers diamond indenter device, this method was used to determine the surface microhardness values. The baseline EMH values for all samples ranged from 339.87 to 352.73, which fall within the literature-reported range for the sound enamel specimens of 250 and 360 VHN [ 6 ]. The EMH values significantly decreased after the induction of initial caries lesions as a result of enamel mineral loss. The SDF-treated group resulted in the highest recovery in the EMH scores (52.43±2.58), followed by the group treated with PVP-coated AgNPs containing varnish (43.21±1.73), and the FV (35.60±1.36). *In vitro* studies on nanosilver fluoride have reported varied findings on its remineralizing potential compared to SDF and FV in artificial caries models, likely due to differences in nanosilver fluoride formulations, experimental conditions, and outcome measures [ 15 - 16 , 18 ]. Nozari *et al* . [ 15 ] found that nanosilver fluoride exhibited superior remineralization efficacy compared to FV and nanohydroxyapatite in primary teeth, possibly due to its optimized silver nanoparticle size (20–30 nm) enhancing fluoride synergy. Conversely, Akyildiz and Sönmez [ 16 ] reported that FV (229.96 VHN) and SDF (222.96 VHN) outperformed nanosilver fluoride (191.36 VHN) in post-demineralization enamel microhardness, suggesting nanosilver fluoride’s limited efficacy, potentially attributable to lower silver content or less favorable pH conditions in their model. El-Desouky *et al* . [ 18 ] indicated that nanosilver fluoride was comparable to FV in limiting enamel demineralization under acidic challenge, likely due to similar fluoride delivery mechanisms, positioning nanosilver fluoride as a potential alternative. Our results, showing modified FV with PVP-coated AgNPs achieving higher %REM than FV but lower than SDF, align partially with Nozari *et al* . [ 15 ] in demonstrating enhanced remineralization over FV, but contrast with Akyildiz and Sönmez [ 16 ] and El-Desouky *et al* . [ 18 ], possibly due to our use of PVP-coated AgNPs, which enhance stability and fluoride interaction. Direct comparisons remain limited, as our study is the first to evaluate PVP-coated AgNPs integrated into FV for primary teeth, offering a novel formulation that balances remineralization and esthetic outcomes. The primary mechanism by which SDF increases hardness is through the formation of silver phosphate (Ag_3_PO_4_), calcium fluoride (CaF_2_), and fluorapatite [Ca_10_(PO_4_)_6_F_2_] on treated surfaces [ 6 ]. Of note, 38% SDF comprises 24–27% silver, 8.5–10% ammonia, and 5–6 % fluoride with a highly alkaline pH of 12.5 [ 6 ]. The concentration of fluoride in 38% SDF (44,800 ppm) is almost double that of FV (22600 ppm). Higher fluoride concentrations result in a more remarkable amount of CaF_2_ formation [ 24 ]. Moreover, CaF_2_ is less soluble in the alkaline pH of SDF, enabling it to serve as a fluoride source for future caries challenges [ 24 ]. The higher %REM values in the samples treated with modified FV with PVP-coated AgNPs than conventional FV might be attributed to the synergistic effect of fluoride and AgNPs [ 15 , 25 - 27 ] along with high silver content [ 24 ]. 

The FESEM observations revealed loss of normal enamel integrity on the demineralized surfaces (Figure 1, b). Treatments with FV, SDF, and FV with PVP-coated AgNPs facilitated the deposition of a mineralized layer on the demineralized surfaces, suggesting enamel repair. Mineral precipitates, fluoride, and/or silver-containing compounds partially occupied the porous areas and defects, leading to relatively recovered enamel surfaces, as shown in Figure 1, c-e. These findings reaffirmed the results of microhardness alteration, which can be attributed to the deposition of a newly formed mineralized layer.

Table 2 presents the EDS results for the elemental compositions of Ag, F, Ca, P, and the Ca/P ratio for the sound, demineralized, and remineralized enamel in different groups. The Ca/P ratio in sound enamel was 2.08±0.004, which decreased to 1.48±0.004 due to mineral diminution after demineralization. These findings are consistent with previous studies [ 20 , 28 ] and the EMH results. Rafiee *et al* . [ 20 ] reported a similar decrease in Ca/P ratio in demineralized primary tooth enamel subjected to a pH cycling model, attributing it to the loss of calcium and phosphate ions. Likewise, Poorni *et al* . [ 28 ] observed a reduced Ca/P ratio after enamel exposure to acidic conditions, linking it to compromised mineral content. The changes in the Ca/P ratio following the application of FV, SDF, and FV with PVP-coated AgNPs confirmed their remineralization potential. Therefore, it is reasonable to suggest that the availability of an extra source for ions can enhance enamel mineral recovery. A Ca/P ratio of 1.6 can lead to optimal enamel remineralization and mineral supersaturation [ 29 ]. Notably, SDF-treated samples could recover the Ca and P values to levels comparable to the sound enamel. The increased Ca/P ratio in all study groups indicated improved resistance against future demineralization challenges. The EDS findings suggest that the SDF-treated surfaces had the highest F and Ag content among all intervention groups, which can be justified by the higher fluoride and silver content in SDF than FV with PVP-coated AgNPs. The EDS mapping demonstrated a consistent distribution of the elements on the treated enamel surface (Figure 2).

The color measurements between the intervention groups revealed noticeable staining in SDF-treated samples, shown by negative ∆L values. Significant differences in ΔE and ΔL values were observed across all groups post-intervention, both in comparison of intervention to demineralization and intervention to baseline stages. Despite the statistically significant difference between the groups, it is important to note that ∆E values exceeding 3.3 correspond to clinically visible changes in tooth color [ 6 ]. Besides, the literature defines the threshold for a clinically acceptable deviation as having a ΔE value of ≤ 6.8 [ 30 ]. Consequently, ∆E _Int-Base_ values for enamel samples treated with modified FV with PVP-coated AgNPs were clinically imperceptible (∆E _Int-Base_= 3±0.325), and ∆E _Int-Dem_ values were within the clinically acceptable range (∆E _Int-Dem_= 6.67±0.49). The higher values of ∆E _Int-Dem_ compared to ∆E _Int-Base_ can be attributed to the more porous structure of demineralized enamel, which can absorb silver ions to a greater extent than the dense crystal structure of the sound enamel [ 31 ]. The discoloration due to SDF would not be clinically acceptable [ 30 ], as it exceeded the acceptable threshold by more than three times. These findings are consistent with Espíndola-Castro *et al* . [ 23 ], who reported pronounced dentin staining with SDF. Similarly, Rafiee *et al* . [ 6 ] noted significant color changes in SDF-treated primary tooth enamel, attributing the black staining to the interaction of high silver content (24–27%) with hydroxyapatite. The black staining is attributed to the deposition of metallic silver. Silver nitrate solution rapidly interacts with hydroxyapatite to form silver phosphate (Ag_3_PO_4_) precipitate, which degrades to black metallic silver when exposed to light [ 31 ]. AgNPs, however, have not been reported to cause color change [ 8 , 23 , 32 ]. Color measurements in the Albahoth *et al* . study [ 33 ] also revealed significant SDF staining, consistent with our findings. According to their findings, nanosilver fluoride with/without L-arginine showed minimal discoloration. They concluded that despite containing silver, nanosilver fluoride’s nano-sized particles (20-30nm) reduce discoloration by minimizing oxidation and precipitation compared to SDF’s larger silver particles [ 33 ]. We propose that the PVP coating on AgNPs prevents enamel surface discoloration by providing a stable, protective layer around the nanoparticles. This coating prevents direct interaction between the AgNPs and the enamel, thereby reducing the intensity of the potential color change due to the presence of silver. The application of capping agents has been demonstrated to be an effective method for stabilizing nanoparticles by different mechanisms such as electrostatic stabilization, steric stabilization, hydration forces, depletion, and van der Waals forces. PVP shows a strong affinity to silver due to its nitrogen and oxygen atoms, acting both as a reducing and capping agent via the steric and electrostatic stabilization of amide groups within the pyrrolidone rings [ 11 ]. This stability might assist in maintaining the nanoparticles' optical characteristics, leading to less visible discoloration on the enamel surface. Compared to FV, which showed minimal color change in a previous study [ 4 ], the present modified FV offers a dual advantage of effective remineralization and esthetic preservation, making it a promising alternative for caries management in primary teeth where esthetic outcomes are critical.

The principle of enamel remineralization involves replenishing lost minerals and sealing demineralized pores by utilizing an appropriate medium to deliver remineralizing agents into the tooth structure. This process aims to reinforce the enamel, restoring its integrity and strength [ 2 ]. Since enamel is a non-living tissue, it cannot naturally remineralize. Thus, the development of remineralizing materials is essential [ 2 ]. Our study assessed the remineralizing potential of modified FV with AgNPs on demineralized enamel surfaces. Despite the constraints imposed by *in vitro* studies, we adhered strictly to the manufacturer's instructions for materials application. We simulated the oral cavity conditions and dynamic pH changes through thermocycling-aging and pH cycling. We restored the samples in deionized water for two weeks, based on the recommendation that caries lesions begin to arrest and reharden within this period after SDF application [ 34 ]. It is important to note that the *in vitro* study may not entirely reflect the complexities of in vivo conditions. Factors such as biofilm formation, the presence of diverse oral flora, different salivary components, individual dietary habits, and oral hygiene practices can influence the effectiveness of remineralization treatments and should be considered when interpreting the results. Therefore, further clinical studies are strongly recommended, especially in primary dentition. 

## Conclusion

The modified FV with PVP-coated AgNPs has the potential to restore the defective enamel by providing an environmentally facilitating condition for newly formed minerals to precipitate. All treatment groups showed improved microhardness values compared to the demineralized enamel. SDF-treated surfaces demonstrated the best remineralization effect. Our findings confirmed that silver particles promote mineral deposition on enamel surfaces. Additionally, PVP-coated AgNPs could effectively prevent the discoloration caused by silver reduction. The slight changes in ∆E values for enamel samples treated with modified FV with PVP-coated AgNPs were clinically imperceptible and considered acceptable. 
